# *In Situ* Hybridisation Study of Neuronal Neuropeptides Expression in Models of Mandibular Denervation with or without Inflammation: Injury Dependant Neuropeptide Plasticity

**DOI:** 10.4172/2157-7099.1000509

**Published:** 2018-06-29

**Authors:** Seham A Abd El-Aleem, Begonia M Morales-Aza

**Affiliations:** 1 Department of Histology, Minia Faculty of Medicine, Egypt; 2 Department of Physiology, School of Medical Sciences, University of Bristol, Bristol, UK

**Keywords:** Neuropeptides, Neurogenic inflammation, Denervation, Inflammation, Periodontitis

## Abstract

Neuronal expression of neuropeptides is altered following peripheral tissue injury associated with inflammation or nerve injury. This results in neuropathic pain with or without neurogenic inflammation which is a major health problem regularly seen in trigeminal neuralgia. Activation of the trigeminal system results in the release of vasoactive neuropeptides substance P and Calcitonin Gene-related Peptide (CGRP) which contribute to nociception, pain and neurogenic inflammation in injured tissues.

**Aim:**

To study the alterations in the neuronal neuropeptides expressions in models of tissue injury associated with either nerve injury or with inflammation and to determine if denervation would alter the neuronal response to inflammation.

**Material and methods:**

Experiments were performed on rat mandibles to produce three models. Firstly, denervation model by sectioning one of the mandibular nerve branches (inferior alveolar nerve). Secondarily, inflammation model by intra-gingival injection of lipopolysaccharide (LPS). Thirdly, combined denervation and inflammation model by sectioning the nerve with subsequent LPS injection. The animals were sacrificed seven days postoperative. Trigeminal ganglia on the operated sides were processed for *in situ* hybridisation for neuropeptides; substance P and CGRP mRNAs. Images were analysed for morphological and morphometric analysis using Image J software.

**Results:**

substance P and CGRP mRNAs were expressed in small and medium-size primary afferent neurons in the mandibular division of the trigeminal ganglia. Both the denervation and the inflammation models showed alteration in neuropeptides expression in the sensory primary afferent neurons innervating the affected mandibular tissues. While, denervation resulted in a significant (substance P=P<0.04, CGRP=P<0.01) downregulation contrarily, inflammation resulted in a significant (P<0.001) upregulation of neuropeptides’ mRNAs. Interestingly, denervation prior to induction of inflammation resulted in insignificant changes in neuropeptides levels. There was a strong correlation (Pearson Correlation=0.8) between substance P and CGRP expression.

**Conclusion:**

We show that tissue damage associated with nerve injury or inflammation results in alteration of neuropeptides levels in the innervating primary afferent neurons. Tissue destruction associated with chronic inflammatory condition such as arthritis and periodontitis are believed to be due to the production of neuromodulators causing neurogenic inflammation. Here we show that denervation abolishes the neuronal response to inflammation. Therefore, tissues denervation could relieve neurogenic inflammation associated with chronic disorders through regulation of neuronal neuropeptide production. Moreover, the current model that combined denervation and inflammation provides a useful animal model to study the contribution of nerve-related mediators in the pathophysiology of tissue injury.

## Introduction

Nervous system plays an important role in the regulation of nociception and pain associated with peripheral tissue injury [1,2]. Nerves secrete neuropeptides which are neuromodulators involved in signal transmission including substance P and CGRP [3]. Neuropeptides are distributed both in the peripheral and in the central nervous system [4–7] and are also expressed in the peripheral tissues [8–10]. Neuropeptides are synthesized in the ganglion sensory cells and transmitted to the peripheral tissues [1,3]. Therefore, the peripheral expression of neuropeptides is partially attributed to neurotransmission from the neurons.

Peripheral stimuli from the orofacial region are transmitted mainly by the trigeminal nerve with its primary afferent neurons located in the trigeminal ganglion [11,12]. Mandibular nerve a branch of the trigeminal nerve provides somatosensory innervation to the mandibular region and its neurons are in the mandibular division of the trigeminal ganglia [13]. Inferior alveolar nerve a branch of the mandibular nerve innervating mandibular structures gingiva, bone and teeth (periodontium) [13,14].

Sectioning the inferior alveolar nerve has been used as a model for studying the effect of denervation on neuropeptides expression centrally in the neurons and locally on the mandibular tissues in health and disease [15–17]. Inflammation of the mandibular structures such as gingiva, gum and bone is referred to as periodontal disease [18,19]. LPS injection into the animal mandible has been used as a model of inflammation for studying the alteration in neuropeptides locally and centrally.

Substance P and CGRP were expressed in the sensory nerve terminals in the gingivae and the periodontium of different species [20–24]. In the trigeminal ganglia, neurons co-express both substance P and CGRP, where all substance P containing trigeminal ganglia cells were expressing CGRP [4–8]. Moreover, there is a correlation between substance P and CGRP and the interaction between them could modulate pain transmission [25–29]. During inflammation, the expression of substance P and CGRP changes in the trigeminal ganglia neurons [30,31] and in the local nerve terminals innervating the inflamed tissues [21–24]. Therefore, neuropeptides play important parts in homeostasis and repair of the innervated tissues and were associated with trigeminal and trigeminovascular disorders [32–36]. We hypothesize that the alteration in neuronal neuropeptide in response inflammation could be modulated by nerve sectioning and this could be the mechanism of relieving pain by tissue desensitization.

## Material and Methods

All animal procedures conformed to UK legislation and local ethical review. Adult male Wistar rats, (225-250 g) were ordered and housed few days prior to the experiment.

### Denervation by sectioning the inferior alveolar nerve

Animals were anesthetized by Hypnorm 0.3 mg/kg (fentanyl citrate 0.1 mg/kg and fluanisone 3 mg/kg) administered intramuscularly, followed by Diazepam 2.5 mg/kg intraperitoneally. The face of the animal was shaved and an incision was made extending from the angle of the mouth to the ear with care not to injure the parotid gland or the facial nerve.

The mandible was exposed by dissecting through the skin, then through the masseter muscle in between the two branches of the facial nerve running across the muscle. A hole was made in the mandibular canal close to the incisor root to get the nerve out of the canal. The nerve was held up and cut (denervated group), then the muscle and the skin were sutured to close the wound. The same procedure was done for the sham group but without cutting the nerve (sham control group).

### LPS-Induction of mandibular inflammation (Periodontitis)

To induce mandibular inflammation a single dose of 1 μl lipopolysaccharide (LPS: 10 mg/ml in saline) (inflammation group) or vehicle (vehicle control group) was injected intra-gingival between the first and second mandibular molars [17], under anaesthesia as above. Animals were given one low dose subcutaneous injection of nonsteroidal anti-inflammatory (Rimadyl 100μl) to minimize the acute pyrexia resulting from LPS injection. Animals were kept warm until full recovery and were checked for any immediate adverse effects of the LPS injection prior to housing under standard conditions (12-hour light: dark cycle, and fed normal rat pellets).

### Animal grouping and tissue harvesting

Animals were grouped as follow; nerve sectioning (denervation group, 3 rats), LPS injection (inflammation group, 4 rats). Nerve sectioning and LPS injection on the same side (combined denervation with inflammation group 6 rats). Control groups included; (sham control 3 rats), (vehicle control 4 rats) and combined sham with vehicle injection on the same side (sham vehicle control, 3 rats). Seven days postoperative, the animals were killed by decapitation under light halothane anaesthesia. The trigeminal ganglia on the operated sides were removed, embedded in OCT and stored at -80°C for subsequent cryosectioning.

### *In situ* hybridization

*In situ* hybridization was performed, as previously described [25]. Briefly, 35S-labelled cRNA probes were synthesised by in vitro transcription from cDNAs encoding preprotachykinin (substance P) and α-CGRP (CGRP) using SP6 RNA polymerase (Promega, Southampton, UK), 35S-labelled UTP (800 Ci/mmol, Amersham Int., Amersham, UK) and unlabelled UTP (Boehringer, Mannheim, Germany), to a specific activity of 3-5 108 Ci/mmol. 10μm cryostat sections of trigeminal ganglia were mounted on gelatin/poly-L-lysine coated slides, fixed in 4% paraformaldehyde in 0.1 M phosphate buffer for 10 minutes and rinsed three times in 2 SSC (standard saline citrate).

**Figure 1:**
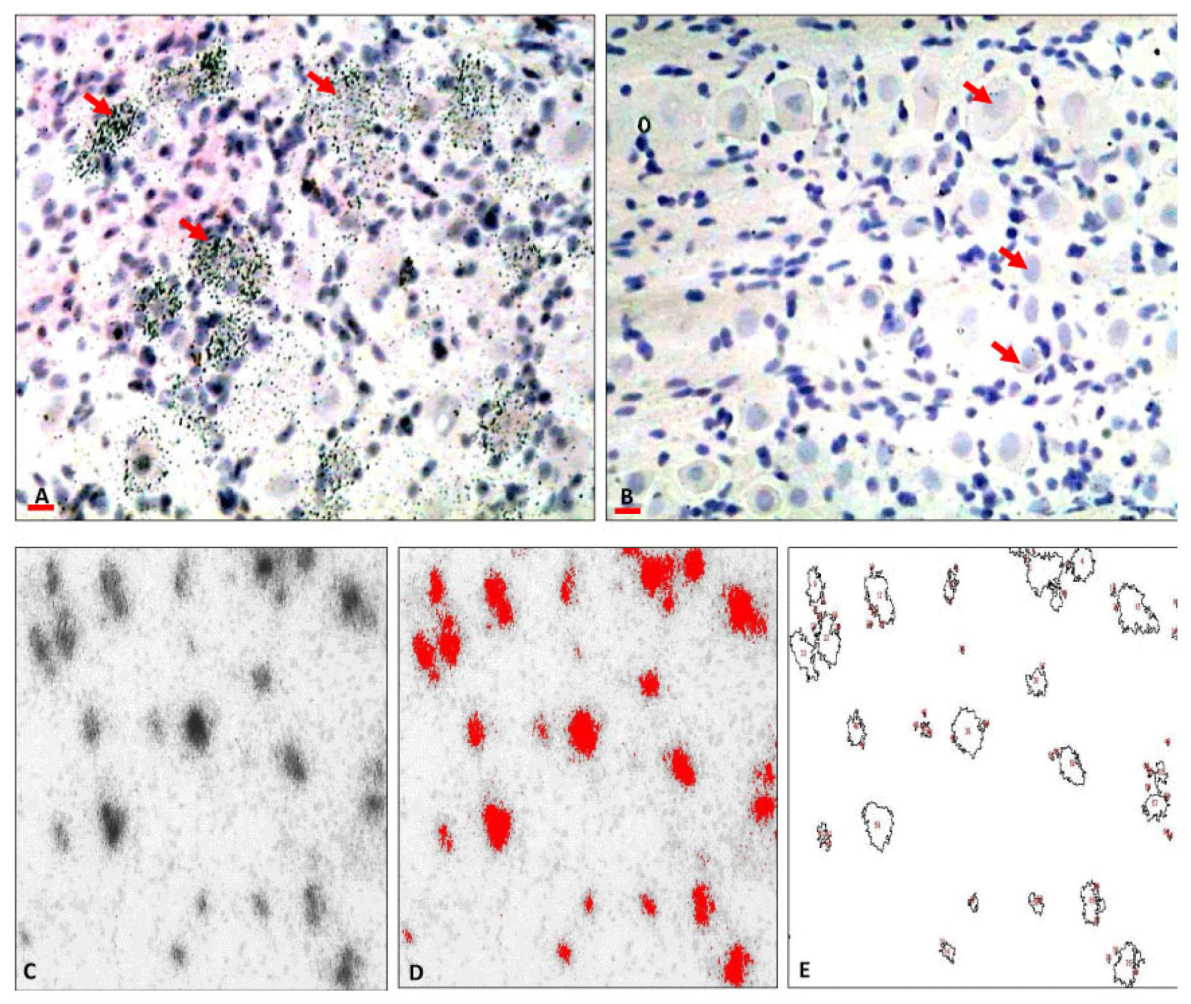
A representative photomicrographs of the trigeminal ganglia showing signal of the *in situ* hybridization staining and the negative control to validate the technique: A) Silver grains (black dots) are seen overlaying the neurons expressing mRNAs. B) Negative sections in which the probe was omitted showing a complete absence of the silver grains. Scale bars: A, B=50 μ. C-E) A representative photomicrograph demonstrating image J analysis of *in situ* hybridisation signals. A) Original image. Silver grains are seen as black dots overlaying the neurons which contain the mRNA. B) Converted image, *in situ* hybridisation signals, were converted into a distinct colour (red) that the software can score. C) Showing the scored neurons (analysed neurons=outlined structures) with exclusion of the background. The analysis provides information about the number of neurons analysed and the levels of the mRNAs per neuron.

Sections were hybridized with approximately 106 counts per ml in hybridization buffer in sealed humid containers at 55°C. After hybridization sections were treated with RNaseA to remove the nonspecific binding then washed to a maximum stringency of 0.1 SSC at 55°C for one hour. Sections were dehydrated in graded ethanols in 0.3 M sodium acetate, air dried and exposed to auto radiographic film. Following film exposure, slides were dipped in K5 nuclear emulsion (Ilford, UK) and exposed at 4°C for up to ten days. Slides were developed, counterstained with hematoxylin and eosin and coverslipped for microscopic examination and image analysis. Negative control sections in which the probe was omitted were processed with each *in situ* run. Sections were examined on a computerized image analysis system (colour still or video camera attached to a Nikon E600 Eclipse attached to Macintosh G4 computer). The positive staining signals (mRNA) were identified by the presence of silver grains (black dots in the bright field) overlaying the cell body of the neurons. Figure 1A and 1B show the staining pattern.

### Image analysis

Autoradiography and computerized image analysis were used to visualize and quantify the hybridization signal. Image J software developed at the U.S. National Institutes of Health was used for the quantification. The levels of mRNA per neuron were determined by counting silver grains overlying cell bodies of neurons [25,26] in the mandibular division of the trigeminal ganglia. The analysis was done for only small size neurons (less than 30 μm diameter) filtered by the software in each of nine individual sections from each animal. The silver grain numbers are represented by the number of the pixels covered by the silver grains for each neuron. Figure 1C-E demonstrates the steps for measuring mRNA levels.

### Data analysis

Statistical analyses were performed using IBM SPSS statistical package. Results were expressed as the mean ± SEM. Expression data were compared using one-way ANOVA comparing the expression levels in each experimental group to those in the corresponding control groups, with P<0.05 being considered as statistically significant.

## Results

### Microscopic examination of neuropeptides expression in control and experimental groups

Neuropeptides; substance P and CGRP mRNAs were expressed in the mandibular division of the trigeminal ganglia from both the control and the experimental groups.

**Figure 2:**
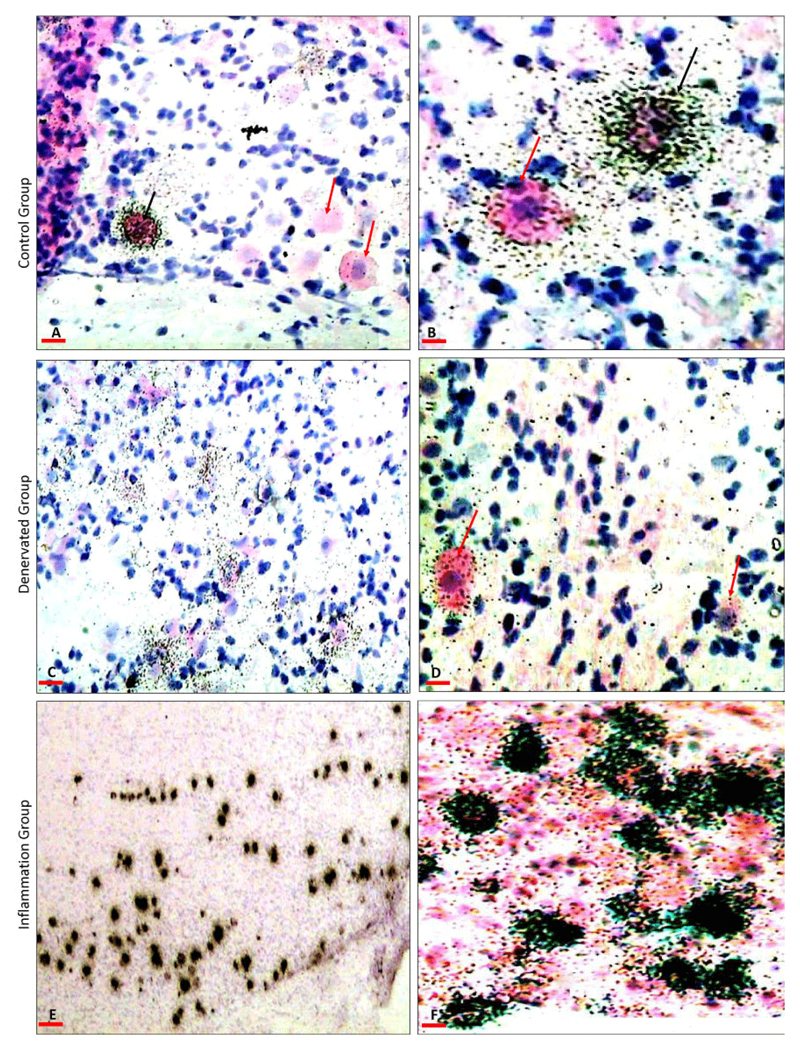
A representative photographs showing substance P mRNA Expression in the mandibular division of the trigeminal ganglia: A) Control trigeminal ganglia showing substance P mRNA expression in neurons only. The positive neuron shows silver grain overlaying the cells (black arrows). B) A higher magnification showing, a variable degree of expression ranging from weak (red arrows) to mild (black arrow). C) Trigeminal ganglia from animals with denervated mandible showing a marked reduction in substance P mRNA in the neurons. Most of the neurons show weak expression. D) Higher magnification showing discrete silver grain overlaying the positive neurons, indicating the low expression (red arrows). E) Trigeminal ganglia from animal with inflamed mandible showing apparent increase in substance P mRNA expression, there is increase in the number of the positive neurons and increase in the expression per neuron. F) A higher magnification showing the coalescence of the silver grains masking the nuclei indicating the marked increase in substance P expression per neuron. Scale bars: A, D, F=50 μ; B=20 μ, C=100 μ, E=400 μ.

**Figure 3:**
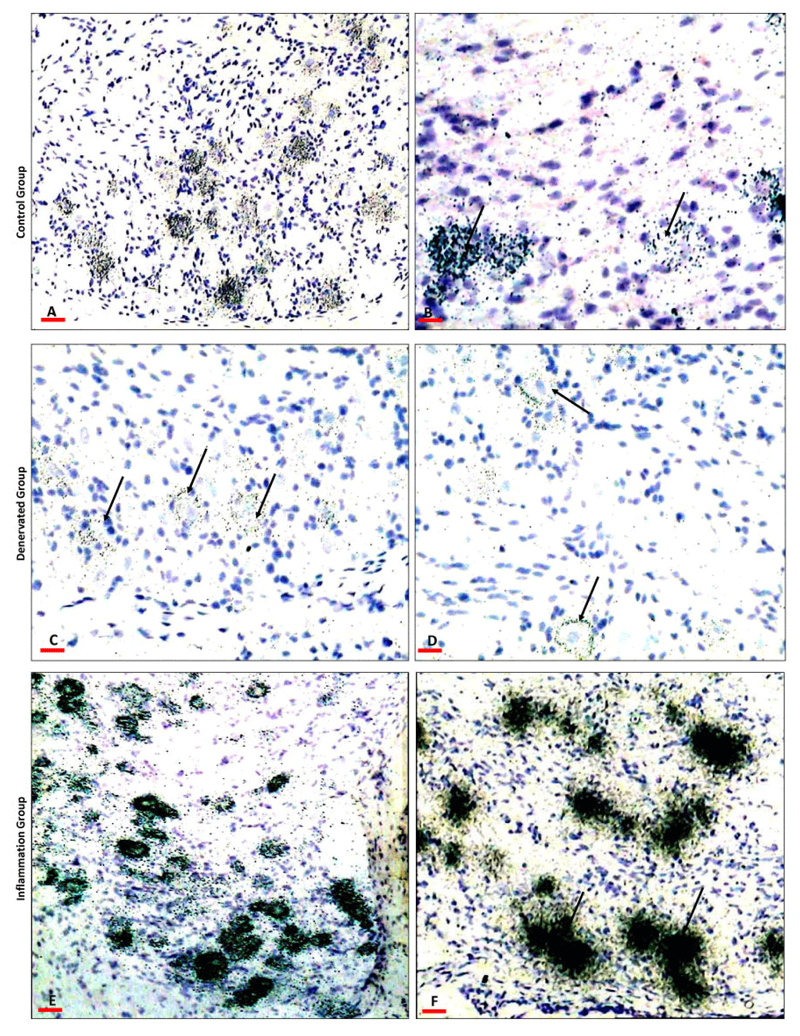
A representative photographs showing CGRP mRNA in the mandibular division of the trigeminal ganglia: A, B) Control trigeminal ganglia, showing CGRP expression in neurons only (arrows). There is wider distribution than substance P as seen in the increase in number of positive neurons. C, D) Trigeminal ganglia from animals with Denervated mandible showing marked reduction in the substance P mRNA in the neurons (arrows). D) Higher magnification showing discrete silver grain distribution overlaying the positive neurons indicating the low expression, the expression was almost limited to the sub membranal cytoplasmic compartment with depletion from the perinuclear cytoplasmic compartment (arrows). E) Trigeminal ganglia from animal with inflamed mandible showing marked increase in CGRP mRNA expression, there is an increase in the number of the positive neurons. F) Higher magnification showing the coalescence of the silver grains masking the nuclei (arrows) indicating the marked increase in CGRP expression per neuron. Scale bars: A=200 μ, B=50 μ, C, D=100 μ, E=300 μ, F=200 μ.

There was variability in the intensity of silver grains indicating variability in the level of expression of neuropeptides in different groups (Figures 2, 3). The expression was exclusively cytoplasmic in the ganglion neurons. Substance P was expressed in small and medium-sized neurons and CGRP was expressed in small, medium and large size neurons.

The quantitation of substance P and CGRP mRNA (Table 1) was performed by a software image J programme which measured the size of neurons and the levels of mRNA per neuron by counting the number of silver grain overlaying the neurons. mRNA levels were measured in the small size neurons (less than 30 μm diameter), which are known to be the sensory neurons associated with nociception.

### Effect of mandibular denervation on the neuropeptides levels in the trigeminal ganglion

**Figure 4:**
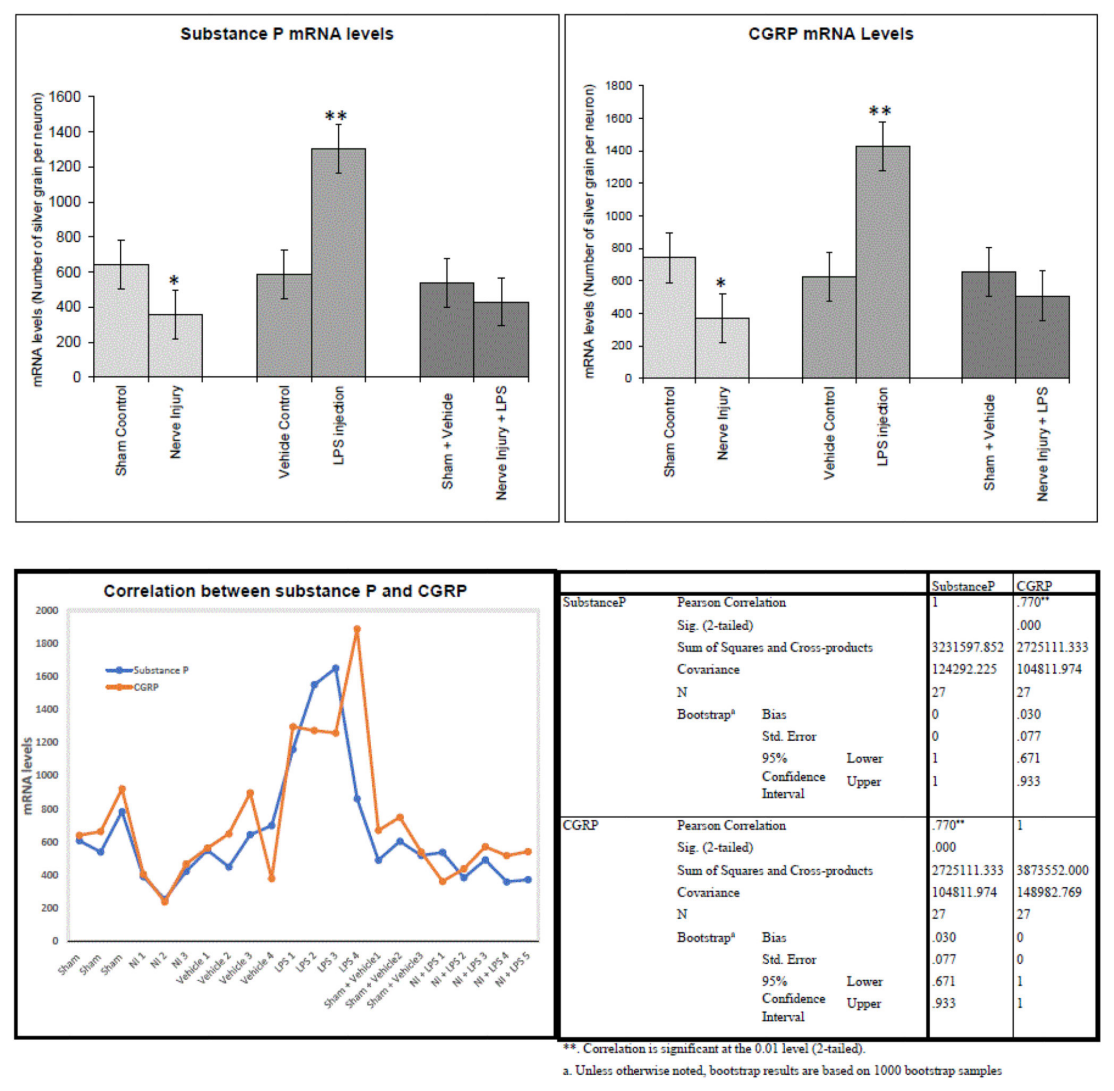
A,B Graphs showing mRNA levels of substance P and CGRP mRNAs in the three groups: Denervated group shows a significant (substance P. P<0.04; CGRP, P<0.01) downregulation by comparison to the control sham group. Contrarily, inflammation group shows a significant (P<0.001) upregulation by comparison to the vehicle control group. Interestingly, denervation of the mandible prior to induction of inflammation abolished the stimulatory effect of LPS on neuropeptides mRNAs. C) A graph showing a strong correlation between substance P and CGRP mRNAs expression (Pearson Correlation=0.8). D) A table summarizing the correlation data between substance P and CGRP.

Sectioning of the inferior alveolar nerve resulted in downregulation in the expression of the neuropeptides; substance P and CGRP in the primary afferent neurons in the mandibular division of the trigeminal ganglia on the operated side. Substance P showed a significant (<0.04) downregulation (Figures 2C, 2D, 4A) by comparison to the control (Figures 2A, 2B, 4A). Similarly, CGRP showed a significant (P<0.01) downregulation (Figures 3C, 3D, 4B) by comparison to the control (Figures 3A, 3B, 4B). Table 1 summarizes the levels of substance P and CGRP in the control and in the denervated groups.

### Effect of LPS induced mandibular inflammation on the neuropeptides levels in the trigeminal ganglia

LPS induced inflammation in the rat mandibular tissues resulted in upregulation in the expression of the neuropeptides; substance P and CGRP in the primary afferent neurons innervating the inflamed mandible (Figures 2-4). Substance P showed a significant (P<0.001) upregulation (Figures 2E, 2F, 4A) by comparison to the control (Figures 2A, 2B, 4A). Similarly, CGRP showed a significant (P<0.001) upregulation (Figures 3E, 3F, 4B) by comparison to the control (Figures 3A, 3B, 4B). Table 1 summarizes the levels of substance P and CGRP in the control and in the LPS injected groups.

### Effect of mandibular denervation and LPS induced inflammation on neuropeptides levels in the trigeminal ganglia

LPS injection in the denervated mandible showed no significant changes in substance P and CGRP expression in the primary afferent neurons in the mandibular division of trigeminal ganglia on the operated side by comparison to the sham-vehicle control group (Figure 4A, 4B). Therefore, nerve sectioning abolished the stimulatory effect of LPS on neuronal neuropeptides production. Table 1 summarizes the levels of substance P and CGRP in the control and in the denervated/inflamed groups. Correlation analysis including all the experimental groups showed a strong correlation (Pearson Correlation=0.8) (Figure 4C) between substance P and CGRP. This indicates that both neuropeptides show a simultaneous change.

## Discussion

Attention has been given to the contribution of the nervous system to inflammatory and healing responses after tissue injury [37]. Several evidences show that neuropeptides such as substance P and CGRP, released from nerve endings, are involved in the host response [38–40]. Different animal models were used to explain the association between the central changes in the innervating neurons and the changes in the local tissues where the nerve ends. These models included either denervation models or inflammation models [40–42]. Here, we have used a denervation model and an inflammation model and we have endorsed our study by using a combined denervation-inflammation model. The three models were used in the same experimental setting, on the same species and under same environmental and experimental conditions to avoid the discrepancies in the results which could result from difference in species, environmental conditions… etc.

Here, we showed that peripheral tissue injury is associated with a concomitant alteration in the neuropeptides centrally in the innervating neurons. While denervation of rat mandible by sectioning one of its branches was associated with downregulation of substance P and CGRP mRNAs in the trigeminal ganglion neurons innervating the affected area. Contrary, induction of mandibular inflammation by injection LPS into the mandible was associated with upregulation of both neuropeptides in the innervating neurons. Interestingly, denervation of the mandible with subsequent induction of inflammation abolished the stimulating effect of LPS and did not affect the neuropeptides production in the innervating neurons. This data adds further evidence that peripheral tissue injury is associated with alteration in neuropeptides expression in sensory neurons and that the nervous system contributes to the tissue inflammation through neuropeptides production and release peripherally [41–48]. Additionally, there is plasticity in neuronal neuropeptides expression which is injury dependent.

The denervation and the inflammation models used in this study showed the alteration in neuropeptides expression centrally. Previous studies using either model have shown similar changes peripherally in the local tissues [41–43]. Denervation models resulted in downregulation of neuropeptide locally in the periodontal bone [41,42] and LPS induced inflammation models resulted in upregulation of neuropeptides in the periodontal bone [42]. Peripheral nerve injury in the ankle joint reduced the expression of substance P and CGRP, while peripheral inflammation at the joint induced upregulation [44–48]. Similarly, in ferret trigeminal ganglia, the expression of substance P and CGRP was downregulated after sectioning the inferior alveolar nerve [17].

Neuropeptides are synthesized in the neurons and transported along the axons to the periphery [49]. Therefore, the local release of neuropeptides from peripheral nerve endings contributes to the inflammation directly by causing vasodilatation and increase vascular permeability resulting in neurogenic inflammation [2]. Moreover, neuropeptides contribute to the inflammation indirectly through the release of other inflammatory mediators such as nitric oxide, prostaglandins and collagenases from the peripheral terminals. Thus, neuropeptides are the major contributor to neurogenic inflammation with subsequent tissue destruction [49].

Sectioning the nerve results in a reduction in the local release of neuropeptides in the peripheral tissues [50–54]. This was attributed to the failure of transport [55] but we have shown here that this is also attributed to the lower neuropeptides production in the neurons. This decrease in neuronal neuropeptides production could be attributed to the retrograde neuronal degeneration following tissue denervation [45] which results in the reduction of the number of neurons in the trigeminal ganglia [50,56]. Additionally, nerve sectioning results in loss of the noxious stimulus for neuropeptides synthesis in trigeminal ganglia [57].

Indeed, sensory neuron plasticity following peripheral tissue injury is considered important for understanding the development of chronic persistent pain [46,47]. This data was further confirmed in the combined denervation-inflammation model when mandibular denervation prior to LPS abolished the stimulating effect of LPS on neuronal neuropeptides production. Therefore, there is a neuronal circuit between the peripheral tissues and the innervating neurons, this account for the concomitant neuronal and local tissue responses to injury. In chronic disorder such as arthritis and periodontitis, the noxious stimulus is transmitted along the nerves causing excessive neuronal neuropeptide production [58]. The neuropeptides are transmitted along the nerves to the periphery and released locally from the nerve ending [59] causing further tissue destruction. Under this condition, denervation would interrupt this circus and minimize the pain and the tissue destruction [60].

In conclusion, neuropathic pain occurs because of tissue injury induced by inflammation or by nerve injury. This is associated with neuronal plasticity. The injury and the intense noxious stimuli stimulate nociceptive neurons. Furthermore, trigeminal nerve injury causes marked plasticity in neuropeptides production in the trigeminal ganglia suggesting that these neuropeptides may play a crucial role in the pathogenesis of orofacial neuropathic pain, in response to peripheral injury.

## Figures and Tables

**Table 1:** Changes in the levels of substance P and CGRP mRNAs in the primary afferent neurons of the mandibular divisions of the trigeminal ganglia on the operated side. Readings represent the number of silver grains (mRNA) per neuron expressed as mean ± SEM.

Groups	Substance P	CGRP
Sham Control	644 ± 73	741.9 ± 90
Denervated	356 ± 40*	371.3 ± 53*
Vehicle Control	586.6 ± 49	623 ± 133
Inflammation	1305.3 ± 163**	1429.5 ± 137**
Sham+Vehicle	538.3 ± 27	653.7 ± 47
Denervation and inflammation	429.8 ± 36	508.7 ± 38

*P value<0.05, **P<0.001
